# Corneal Epithelial Stem Cells–Physiology, Pathophysiology and Therapeutic Options

**DOI:** 10.3390/cells10092302

**Published:** 2021-09-03

**Authors:** Yue Ruan, Subao Jiang, Aytan Musayeva, Norbert Pfeiffer, Adrian Gericke

**Affiliations:** 1 Department of Ophthalmology, University Medical Center, Johannes Gutenberg University Mainz, Langenbeckstrasse 1, 55131 Mainz, Germany; sjiang@uni-mainz.de (S.J.); norbert.pfeiffer@unimedizin-mainz.de (N.P.); 2 Laboratory of Corneal Immunology, Transplantation and Regeneration, Schepens Eye Research Institute, Massachusetts Eye and Ear, Department of Ophthalmology, Harvard Medical School, Boston, MA 02114, USA; aytan_musayeva@meei.harvard.edu

**Keywords:** cornea, epithelium, stem cell deficiency, graft, limbus, niche, transplantation

## Abstract

In the human cornea, regeneration of the epithelium is regulated by the stem cell reservoir of the limbus, which is the marginal region of the cornea representing the anatomical and functional border between the corneal and conjunctival epithelium. In support of this concept, extensive limbal damage, e.g., by chemical or thermal injury, inflammation, or surgery, may induce limbal stem cell deficiency (LSCD) leading to vascularization and opacification of the cornea and eventually vision loss. These acquired forms of limbal stem cell deficiency may occur uni- or bilaterally, which is important for the choice of treatment. Moreover, a variety of inherited diseases, such as congenital aniridia or dyskeratosis congenita, are characterized by LSCD typically occurring bilaterally. Several techniques of autologous and allogenic stem cell transplantation have been established. The limbus can be restored by transplantation of whole limbal grafts, small limbal biopsies or by ex vivo-expanded limbal cells. In this review, the physiology of the corneal epithelium, the pathophysiology of LSCD, and the therapeutic options will be presented.

## 1. Introduction

In 1868, the term “stem cell” was first proposed in the worldwide scientific literature by the German biologist Ernst Haeckel [1]. Stem cells are unspecialized cells with differentiation potential that can simultaneously self-renew, differentiate and generate any cell type in the organism, with slow cycling during homeostasis in vivo [2,3]. Over the years, stem cells became attractive for therapeutic applications in many branches of medicine, and a growing amount of knowledge is being gathered in this field [2]. Based on their differentiation potential, stem cells can be classified into totipotent, pluripotent, multipotent, oligopotent, and unipotent stem cells [2]. Moreover, according to the stage of development, stem cells can be divided into embryonic and adult stem cells [4]. Embryonic stem cells are pluripotent and derived from blastocysts [5]. Adult stem cells exist in adult tissues and have less differentiation potential than embryonic stem cells [6]. However, some adult forms of stem cells are thought to possess multipotent, oligopotent, and unipotent properties [4,7]. Some adult stem cell types, such as bone marrow and dental pulp stem cells, are believed to even possess pluripotent properties [8,9]. Under physiological conditions, adult stem cells, which can create progenitor cells, are usually quiescent [10,11]. However, adult stem cells can be reactivated to proliferate for tissue regeneration under various extrinsic stimuli, such as stress and injury [12]. In 1971, Davanger and Evensen proposed that renewal of the corneal epithelium is driven by migration of epithelial cells located at the limbus of the cornea [13]. The hypothesis of limbal epithelial stem cells (LESCs) has been studied and proven in various in vivo and in vitro studies [14,15,16]. Moreover, there is some evidence for stem-like and progenitor cells in the peripheral corneal endothelium [17,18]. Furthermore, small populations of stem cells in the human corneal stroma have been identified [19]. Taken together, not only LESCs but also corneal stromal stem cells and endothelial stem cells appear to exist in the cornea, which are all considered to play a vital role in maintaining corneal homeostasis and repair [20,21,22,23].

The cornea is a transparent and refractive structure at the front of the eye that transmits and focuses light to the retina and lacks blood vessels [24]. Hence, loss of corneal transparency results in severe visual impairment or blindness [25]. The World Health Organization estimates that around 4.2 million worldwide are suffering from moderate to severe distance vision loss or blindness due to corneal opacities, which has become the fourth leading cause of visual impairment [26].There is a wide variety of corneal diseases that can cause corneal opacification or deformation, e.g., corneal trauma, microbial infection, neurotrophic keratopathy, endothelial dystrophy, or limbal stem cell deficiency, which can be acquired or inherited [27,28,29,30]. 

Various corneal transplantation techniques have been developed to treat loss of corneal transparency in cases where conservative strategies are unavailable or unsuccessful [31]. Due to the relative immune privilege of the cornea, the prognosis of corneal grafts is relatively good [32]. However, transplantation of epithelial stem cells is challenging, since they are believed to be localized at the densely vascularized limbal region, which is highly immunogenic [33]. Therefore, a variety of conservative strategies is under investigation to treat persistent corneal epithelial defects and limbal stem cell disease.

This review summarizes the location, physiology, and pathophysiology of corneal epithelial stem cells, and provides a view on the recent significant findings of clinically established therapies for stem-cell diseases. We also discuss novel potential treatments aimed at improving corneal regeneration.

## 2. Localization of Limbal Epithelial Stem Cells

The cornea is composed of five layers: the epithelium, Bowman’s layer, stroma, Descemet’s membrane, and the endothelium, and each of those layers exerts an essential role in maintaining corneal transparency and stable visual function [34]. In 2013, Dua et al. proposed a novel sixth layer in the human cornea located between the stroma and Descemet’s membrane [35]. The authors suggested the terms “Dua’s layer”, “pre-Descemet’s layer (PDL)” and “pre-posterior limiting lamina layer” for this anatomical part of the cornea [35,36]. Although there are still many controversies about this anatomical structure, it already had a significant impact on corneal research and therapy [35,36,37,38,39,40]. Clear vision requires a concertedly functional cooperation of the individual corneal layers to maintain a smooth and transparent corneal surface [33]. The corneal epithelium is the cornea’s outermost layer with five to seven cell layers that are together 50 to 60 μm thick [15,34,41]. It consists of inner basal cells, middle wing cells and superficial squamous cells [34]. The limbal corneal epithelium is composed of several layers of epithelial cells with Langerhans cells and melanocytes [15]. The role of the corneal epithelium includes maintenance of corneal transparency and protection of the eye from the external environment [42]. 

**Figure 1:**
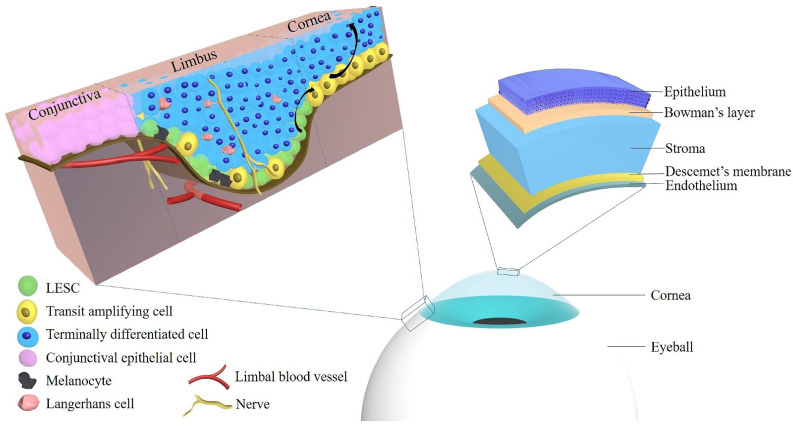
The localization of limbal epithelial stem cells and the anatomy of the cornea. Abbreviations: LESC: limbal epithelial stem cell.

In 1983, Thoft and Friend proposed the X, Y, Z hypothesis of corneal epithelial maintenance [43]. The authors suggested that proliferation of corneal basal epithelial cells (X) and centripetal movement of peripheral cells (Y) equals the epithelial cell loss from the corneal surface (Z) so that cell replacement may maintain and replace cell loss in the cornea [43]. These peripheral cells reside exclusively in the limbal zone and simultaneously maintain a steady cell number by giving rise to transit amplifying cells (TAC) [44]. By a scanning slit confocal microscope, investigators tested the hypothesis that cells migrate centripetally in the epithelial layer of the normal human anterior cornea [45]. However, the origin of these corneal epithelial cells was not described in the X, Y, Z hypothesis in 1983. Thoft presented another assumption later that conjunctival cells in the limbus could supply peripheral corneal cells by crossing the limbus, which has been termed “conjunctival transdifferentiation” theory [46]. In 1986, Schermer et al. used a new monoclonal antibody, AE5, in vivo and in culture to recognize a 64-kDa basic (K3) keratin, a specific corneal epithelial differentiation marker in an advanced stage of basal epithelial cells of the central cornea, but not in conjunctival cells [47]. Therefore, Schermer et al. considered that not conjunctival cells, but corneal epithelial cells, are responsible for the maintenance of the corneal epithelium, and they suggested that corneal epithelial stem cells are located at the limbus, a zone between the cornea and the conjunctiva [47]. By using the monoclonal antibody, 4G10.3, directed against a 50-kD protein and binding to the limbal basal cells, Chung et al. studied the localization of corneal epithelial stem cells in rats during development in life (from day one to six weeks of age) [48]. The authors suggested that stem cells or stem-like cells are localized throughout the basal layer of the corneal and limbal epithelium from day 1 through the whole adult life [48]. These epithelial stem cells are sequestered in the limbus when the cornea matures [48]. Two positive (p63, CK14) and one negative stem cell marker (CK3) have been used to identify the location of corneal epithelial stem cells in horses [49]. The results revealed that equine corneal epithelial stem cells are localized in the epithelial basal layer of the corneal limbus, which is rich in resident melanocytes. Hence, melanocytes might serve as an indicator of the collecting area of corneal epithelial stem cells [49]. Moreover, a member of the ATP binding cassette (ABC) transporters, ABCG2, was shown to be expressed by limbal basal cells [50]. In a rabbit model, in which the limbal epithelium was surgically removed, recurrent erosions and corneal vascularization with delayed healing occurred whereas the controls healed normally [16]. These signs could be related to limbal stem cell dysfunction [16]. Figure 1 shows the anatomy of the cornea and limbus and the localization of LESCs.

**Figure 2:**
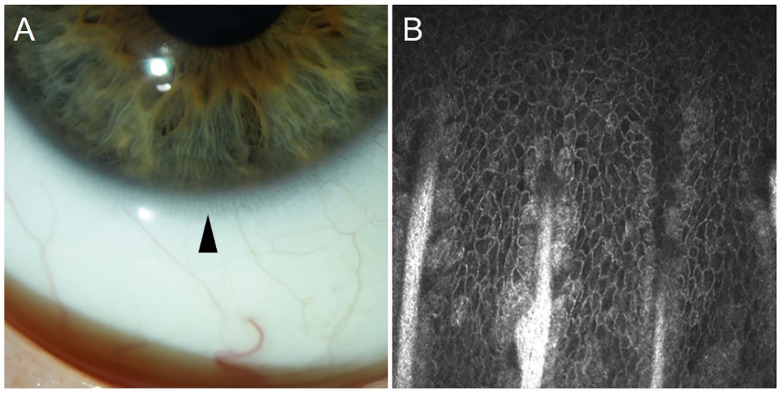
The limbal palisades of Vogt taken by slit lamp (arrowhead, (**A**)) and by in vivo confocal microscopy (Heidelberg Engineering GmbH, Heidelberg, (**B**)).

Pellegrini et al. proposed that the location of stem cells can be strongly supported by the evaluation of their cell division capacity, even though some corneal differentiation markers for locating epithelial stem cells have been reported [15]. By serially cultivating cells from the ocular surface, Pellegrini et al. found that the cells from nine limbal biopsies underwent 85 doublings, whereas cells from central cornea could not be serially cultivated, indicating that cells in the limbal region of the corneal epithelium have a high proliferative capacity, indicative of stem cell existence [15]. Ebato et al. compared the mitotic rate of cells from the corneal limbus, the peripheral epithelium, and the central epithelium in tissue culture [51,52]. The results indicated that the mitotic rate of outgrowths of limbal cells was significantly higher than that of peripheral epithelium, which in turn was higher than that of the central epithelium [51,52]. Altogether, the existing data strongly suggest that corneal epithelial stem cells reside in the basal layer of the corneal limbal epithelium [53]. The stem cells are believed to be especially rich at the limbal palisades of Vogt, which are radially oriented fibrovascular ridges especially concentrated along the superior and inferior limbus [54]. Images of Vogt palisades taken by slit lamp and in vivo confocal microscopy are depicted in Figure 2.

Based on studies using in vivo confocal microscopy and three-dimensional (3D) imaging, Shortt et al. modeled the regional distribution and structure of the corneal epithelial stem cell niche, and indicated how epithelial stem cells can be located and observed in vivo in humans [55]. Their results suggested that corneal epithelial stem cells line the edges and bases of limbal crypts and are also located around the sides and tips of focal stromal projections, surrounded by a complex vascular plexus [55]. Their findings reveal three different stem cell niches: limbal epithelial crypts, limbal crypts, and focal stromal projections [55]. The limbal crypts are most densely distributed in the superior and inferior limbal quadrants [55].

## 3. Limbal Epithelial Stem Cell Markers

Several potential markers have been used to identify LESCs, which will target the emerging therapeutic topics. It is therefore useful to summarize these markers of LESCs.

### 3.1. p63

The p63 gene produces full-length (TAp63) and N-terminally truncated (ΔNp63) transcripts that can be spliced to encode three different p63 isoforms: p63α, p63β, and p63γ [56,57]. The p63 protein was found in various human tissues and is a transcription factor regulating the progression of the cell through its cycle and cell death in response to environmental stimuli [56]. Pellegrini et al. have shown that the nuclear p63 transcription factor (ΔNp63α), a p53 homolog related to epithelial regenerative proliferation, is expressed by basal cells of the limbal epithelium in vivo and in vitro, but not by TACs [58,59]. The p63β and p63γ isoforms, which are absent in the resting limbus, are inclined to participate in epithelial differentiation during corneal regeneration [60]. Some scientists suggest that evaluation of p63 in cultivated limbal epithelial cell sheets is vital for assessing and selecting the quality of the cell sheet [61]. In addition, p63 knockout mice lack all stratified squamous epithelia and do not express differentiation markers [62]. In a clinical study, Rama et al. cultivated limbal stem cells on fibrin to treat 112 patients with corneal damage [63]. Their results showed that successful transplantation of patients was associated with the percentage of p63-bright holoclone-forming stem cells in culture [63]. Via performing in situ hybridization with [^35^S]-labeled sense and antisense p63α RNA riboprobes, strong p63 mRNA signals were found only in the basal layer of limbal epithelia [64]. 

### 3.2. ABCG2

ABCG2, also known as breast cancer resistance protein 1 (BCRP1), is a member of the ABC family, as a universal marker for stem cells in many tissues [65]. For example, ABCG2/BCRP1 expression is related to the side population (SP) phenotype based on the ability to efflux Hoechst 33342 dye, as a marker for hematopoietic stem cells [65]. The limbal epithelium contains SP cells that express ABCG2, which are considered LESCs [66]. By immunocytochemistry and quantitative PCR, ABCG2 was detected in the basal layer of the limbal epithelium [64,67]. Moreover, ABCG2 mRNA was shown to be expressed at low levels by corneal epithelium and at higher levels by limbal epithelium [50]. As shown by flow cytometry, ABCG2-positive limbal basal cells exhibited greater colony-forming efficiency than corneal epithelium and possessed stem cell properties on a 3T3 fibroblast feeder layer (mouse embryo fibroblast) [50]. In an in vitro study, Kethiri et al. elucidated the ideal limbal explant size and number for limbal transplantation by assessing the expression of ABCG2 [68]. Their findings revealed that a minimum amount of 0.3 mm^2^ vital tissue would be adequate for ample limbal cell expansion, and that larger cadaveric explants (≥0.5 mm^2^) had a similar growth rate and proliferative potential to the live tissue [68]. In an ABCG2 knockout mouse model, cultured corneal epithelial cells lacking ABCG2 had an increased susceptibility to oxidative damage induced by mitoxantrone and hypoxia, suggesting that ABCG2 may protect corneal epithelial cells by exerting anti-oxidative effects [69]. In one study, pieces of the limbus from the donor’s limbal zone have been cultured. From these pieces, ABCG2-positive limbal stem cells could be expanded as cell sheets after three weeks of culturing on amniotic membrane. The cells could also be cryopreserved [70]. 

### 3.3. Growth Factor Receptors

Based on the theory that the epidermal growth factor receptor (EGFR) is localized in various stratified squamous epithelia, Zieske and Wasson studied the growth factor receptor in the rat cornea in 1993 [71]. The authors showed by an antibody binding to the EGFR that staining was more intense in basal cells from the corneal limbal zone [71]. In studies on human ocular surface epithelia, EGFR immunostaining was observed in basal corneal epithelial cells [72]. Keratinocyte growth factor (KGF) is a mesenchymal-cell-derived paracrine growth factor, which is consistently more produced by limbal fibroblasts than by central corneal fibroblasts [73]. KGF stimulates the differentiation, proliferation, and migration of limbal epithelial cells via a KGF paracrine loop, inducing ΔNp63α expression [74]. In a human in vitro limbal epithelial cell model, KGF induced higher expression of the epithelial stem marker, ΔNp63α, compared with epidermal growth factor [74]. Moreover, it has recently been shown that limbal epithelial cells cultured with KGF and the rho kinase inhibitor, Y-27632, could maintain the expression of several LESC markers, which may become an improved in vitro model in regenerative medicine [75]. Furthermore, the keratinocyte growth factor receptor (KGFR) has been mainly expressed on the mRNA level by cultured limbal epithelial cells [76]. 

### 3.4. Integrins

The integrins, consisting of individual α and β subunits, are the primary metazoan receptors that are vital for a metazoan existence and play critical roles in mediating cell adhesion [77]. There are several integrin subunits reported to be expressed in the corneal epithelium, including β1, β4, β5, α2, α3, α5, α6, and αv [78]. The β1 integrin subunit is mainly located at the membranes of basal epithelial cells in the cornea, and anti-β4 immunofluorescence was only shown in the basal part of the epithelium [79]. Pajoohesh-Ganji et al. reported that limbal basal cells at the superior and inferior quadrants of adult mouse corneas expressed higher levels of β1 and β4 integrin and less α9 integrin than limbal basal cells at the nasal and temporal quadrants [80]. Hayashi et al. reported that LESCs could be enriched by integrin α6 and the transferrin receptor (CD71), localized immunohistochemically in the basal region of the limbal epithelium, suggesting that α6 integrin and CD71 are the cell surface markers of LESCs [81]. After mesenchymal stem cell transplantation in an experimental rabbit model of LSCD, expression of β1 integrin and connexin 43 (Cx43) were used as indicators for putative LESCs [82,83]. These studies provided crucial evidence that corneal integrins are potential markers of LESCs and are essential for supporting the function of LESCs [80,84,85,86]. 

### 3.5. Keratins

Keratins are the major structural proteins of epithelia, which play a critical role in the integrity and mechanical stability of epithelial cells and tissues [87]. In addition, several keratins mediate tissue differentiation and participate in intracellular signaling under various pathologic conditions [88]. Like epidermal epithelium, the corneal epithelium contains significant amounts of keratins, such as K3/K12, K5/K14, K5/K12, K8/K18, and K8/K19 [89,90,91]. However, K14 and K5 are not only located at the limbus but also reside in epithelial cells of the corneal center, suggesting that K5/K14 is an unreliable marker for LESCs [92]. The corneal epithelial cells’ intermediate filament cytoskeleton comprises the cornea-specific keratins, K3 and K12 [93]. Additionally, RT-PCR studies revealed lower K3 and K12 mRNA levels in limbal epithelia than in the corneal epithelia [64]. Other studies proved that the limbal basal cells lack the corneal keratins, K3 and K12 [47,94]. During corneal wound healing, K14 was expressed in niches at the limbal area seven days after birth in the mouse cornea [95]. K8 and K19 were present in the limbal niches at day 21 after birth [95]. The corneal epithelium showed enhanced K14 expression by day 28 after wounding, suggesting that LESCs migrate centripetally towards the central cornea [95]. In contrast, the expression of K8 in limbal niches does not change with wound size [96]. In 2019, Park et al. showed that K14^+^ basal epithelia migrate into the wound bed by increased population pressure gradient from the limbus to the wound edge [97]. Thus, the authors demonstrated that LESCs participate in corneal wound healing by using bromodeoxyuridine (BrdU) labeling to visualize their contribution in real-time [97]. Table 1 lists the keratins in different species during the development and injury healing of the cornea. 

## 4. Pathophysiology of Limbal Stem Cell Deficiency

Limbal stem cell deficiency (LSCD) is a progressive corneal epithelial disorder characterized by recurrent erosion, corneal vascularization, and conjunctival epithelial ingrowth, resulting in corneal opacity and visual impairment [100]. Many causes are related to the direct depletion of LESCs and/or destruction of the LESC niche, leading to LSCD that may present unilaterally or bilaterally and be of partial or total extent [101]. Published studies show that chemical and thermal burns accounted for approximately 75 % and ocular surface inflammatory diseases for 7.8% of all reported causes of LSCD [102,103,104]. According to a surveillance study from Australia and New Zealand in 2017, it has been noted that the most common causes of LSCD were contact lens over-wear (accounting for 21%), cicatrizing conjunctivitis (21%), and chemical/thermal injury (14%) [102]. In addition to the etiologies of LSCD above, there is a range of acquired and hereditary LSCD. Inflammatory ocular surface diseases involve Stevens-Johnson syndrome, microbial infection, mucous membrane pemphigoid, and chronic vernal keratoconjunctivitis. Congenital diseases include aniridia, dyskeratosis congenita, epidermolysis bullosa, and epidermal dysplasia, whereas acquired LSCD contain chemotherapy, iatrogenic injury, and ultraviolet irradiation [104,105,106,107,108,109]. One of the most common causes of congenital LSCD is aniridia [104]. Other studies have shown that congenital aniridia had a significantly increased association with glaucoma, which is the most common ocular comorbidity in LSCD [110,111]. Congenital and acquired causes of LSCD are listed in Table 2.

LESCs and the limbal microenvironment are considered to play an essential role in maintaining corneal avascularity and corneal immune privilege as a “barrier” to prevent conjunctivalization and propagation of blood and lymphatic vessels from migrating onto the corneal surface [159]. Hence, depletion of LESCs with the destruction of their stem cell niche may change corneal homeostasis [159]. For example, under pathological conditions, conjunctival epithelia can drift across the limbal margin, at worst causing centripetal insurgence of inflamed fibrovascular conjunctiva [160,161]. Corneal neovascularization is regulated by the vascular endothelial growth factor (VEGF) family of proteins, which is a crucial mediator of embryonic vasculogenesis and angiogenesis, including placenta growth factor (PlGF), VEGF-A, VEGF-B, VEGF-C, and VEGF-D, and the viral VEGF-Es [159,162]. Amano et al. found that after corneal injury, VEGF mRNA and protein were induced to high levels, and they first demonstrated that VEGF might be required for inflammatory neovascularization of the rat cornea [163]. VEGF may regulate the process of corneal conjunctivalization characterized by goblet cells and neovascularization via the VEGF receptor 1 (VEGFR-1) [164]. Furthermore, the cytokines including interleukin (IL)-1β, IL-6, IL-7, IL-8, matrix metalloproteinases, and tumor necrosis factor-alpha were reported to participate in corneal neovascularization and conjunctivalization [165,166,167,168]. IL-6 can amplify inflammatory responses and induce secretion of VEGF from fibroblasts [169]. The conjunctival epithelium is rich in goblet cells and highly vascularized. Hence, it is not surprising that it leads to an inferior optical quality compared to corneal epithelium when reaching the corneal center [165]. Moreover, deposition of lipids in the cornea during LSCD may cause damage to corneal integrity [165]. 

## 5. Therapy of LSCD

**Figure 3:**
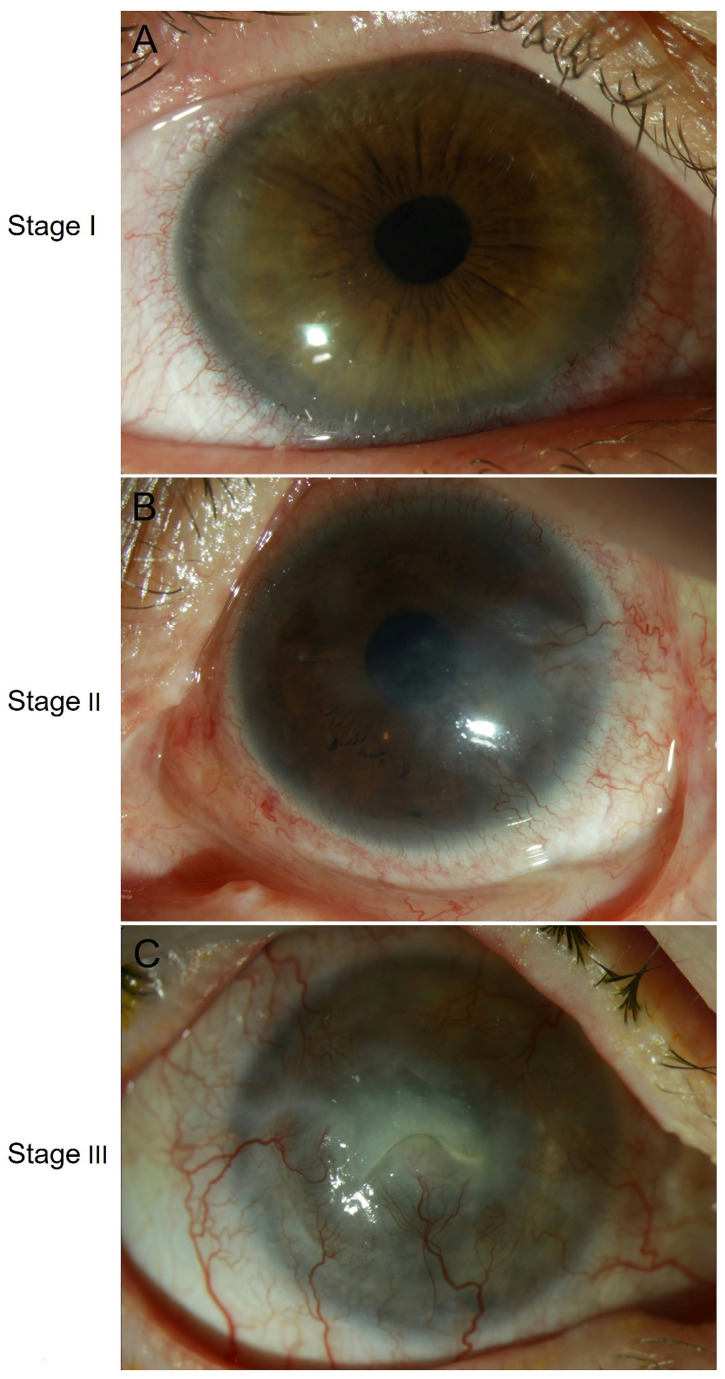
Slit-lamp photographs of eyes with stage I, stage II, and stage III of LSCD, respectively. (**A**) Corneal opacity and corneal pannus not extending to the central 5 mm zone of the cornea with less than 50% of limbal involvement. At the 4 to 6 o’clock positions, conjunctival vessels are growing onto the peripheral cornea, which corresponds to stage I LSCD. (**B**) Corneal opacity affecting the central 5 mm zone of the cornea with less than 50% of limbal involvement corresponding to stage II LSCD. (**C**) Conjunctival growth on the cornea is seen at each limbus position, and the entire cornea is vascularized and opacified. Moreover, a central epithelial defect is seen. This example corresponds to stage III LSCD [171]. Abbreviations: LSCD: limbal stem cell deficiency.

Therapeutic options of LSCD rely upon its etiology, the degree of severity and the laterality of LSCD, which have been recognized as prognostic indicators for recovery [170]. Dua et al. suggested that the prognosis of LSCD depends on the involvement of the limbus (clock-hours) and conjunctiva (percentage). Based on this hypothesis the authors proposed a new classification grading scale for the severity of ocular surface injury [170]. In 2019, the Limbal Stem Cell Working Group published a global consensus statement, which provides a classification system, diagnostic and staging criteria for LSCD staging with an international agreement [171]. According to this global consensus, there are three stages of LSCD based on the clinical presentation: stage I, stage II, stage III [171]. Figure 3 shows representative slit-lamp photographs of different stages of LSCD. Measures for patients with LSCD are very challenging because one treatment approach cannot be applied for all patients. In recent decades, much effort has been put into developing more effective measures for LSCD, including conservative medical therapy, surgical techniques, and innovative transplantation strategies [172]. 

### 5.1. Conservative Therapy

Conservative therapeutic approaches aim to control causative factors, to alleviate pain and to restore a stable ocular surface in the early stage of LSCD, including nonpreserved lubricating eye drops, autologous serum drops, bandage contact lenses, anti-inflammatory drugs, and anti-angiogenic therapy [173,174,175,176,177]. Autologous serum eye drops as a natural preservative-free and growth factor-containing lubricant can promote corneal epithelial healing and medically reverse advanced LSCD [178,179]. Comparing the effect of autologous serum eye drops with different diluents, several studies have shown that higher concentrations (100%) of serum eye drops provide more benefits than lower concentrations in optimizing tear film stability and reducing symptoms [179,180,181,182]. Bandage contact lenses, including rigid gas permeable contact lenses, soft hydrogel contact lenses and silicone hydrogel contact lenses, can improve epithelial healing and eliminate chronic irritation in persistent epithelial defects [183,184]. Topical anti-inflammatory drugs, including non-steroidal anti-inflammatory drugs (NSAID) and ophthalmic steroids, play a role in suppressing the initial inflammatory response and in preventing LSCD [185,186,187]. However, treatments with ocular lubricants and topical steroid drops require long-term and frequent application, which requires adherence from patients and may result in adverse events, such as glaucoma, ocular infection, and cataract [188].

### 5.2. Basic Surgeries and Novel Techniques

When unavoidable destruction of the LESCs presents between the corneal and conjunctival epithelia, the conjunctival epithelium will grow across the “limbal barrier” and cover the cornea with conjunctival epithelial cells, including goblet cells as well as blood and lymphatic vessels, causing conjunctivalization of the corneal surface and leading to vision impairment [159,189]. The conjunctival epithelium covering the cornea is announced to undergo a process termed “conjunctival transdifferentiation”, in which the conjunctival epithelium transforms into a cornea-like epithelium [190]. Some studies have shown that conjunctival transdifferentiation can be induced by deficiency of vitamin A and occlusion of corneal vessels [190,191,192,193]. Dua et al. proposed that it is usually unnecessary to scrape conjunctival epithelium off from the cornea as long as the pupillary area is not covered and the symptoms are tolerable [194]. In clinical practice, sequential sector conjunctival epitheliectomy (SSCE) can significantly improve vision when the pupillary area is covered by conjunctival epithelium [194]. SSCE is performed by repeated brushing or scraping of conjunctival epithelium towards the limbus, until limbus-derived corneal epithelium covers the denuded surface [195]. However, this procedure can cause pain and bleeding to patients by debridement of the fibrovascular pannus, and it requires multiple patient visits for repeated monitoring and treatment until the cornea is fully re-epithelialized [195]. Moreover, amniotic membrane transplantation (AMT) is considered as an alternative treatment for LSCD, greatly owing to the amniotic membrane’s biological properties for providing protection [196,197].

Nevertheless, several clinical trials showed that AMT has advantages in moderate LSCD, but not in severe cases, with no definite advantage of AMT alone over medical therapy [197,198]. The combination of SSCE with AMT appears to be an effective procedure for treating LSCD [199,200]. Notably, Dua et al. developed the original surgical technique named amnion-assisted conjunctival epithelial redirection (ACER), by performing a 360° peritomy and utilizing amniotic membrane to redirect the conjunctiva growing over the amnion, excluding admixture of the corneal and conjunctival epithelium [195]. Moreover, amniotic membrane suspension at concentrations of 15% and 30% may be beneficial for human corneal epithelial cell viability, migration, and proliferation [201]. Bischoff et al. reported that amniotic membrane-conditioned medium containing epithelial growth factor, fibroblast growth factor basic, IL-6, and IL-8 is a nonsurgical treatment alternative for non-healing corneal epithelial defect [202]. Even though amniotic membrane has unique anti-inflammatory, anti-angiogenic, and anti-fibroblastic properties, human amniotic membranes are obtained from elective cesarean section, putting amniotic membrane at potential risk of transmitting diseases and infections if not thoroughly tested [203]. 

Therefore, many techniques have been developed to preserve most of the biological properties of amniotic membranes by storage techniques and to prevent disease transmission to recipients [204,205,206]. In 2021, a cohort study from Queen Victoria Hospital and Maidstone Hospital showed that a dry and stable human amniotic membrane-derived matrix, Omnigen^®^ using OmniLenz^®^ (NuVision Therapies, Nottingham, UK), can easily be applied in the clinical setting with biochemical stability and efficiency as a novel tool to treat LSCD [207,208]. Based on the technique of dried amniotic membrane, Ting et al. developed a modified ACER method by using low-temperature vacuum-dehydrated amniotic membrane instead of the cryopreserved amniotic membrane and fibrin glue rather than sutures [206,209]. In 2021, another modified ACER technique was introduced, which was combined with superficial keratectomy for the treatment of partial LSCD [210]. The medical records of patients with partial LSCD showed that the method successfully prevented the invasion of conjunctival epithelial cells into the cornea, and the corneal surface was re-epithelized by corneal epithelial cells derived from the remaining LESCs [210]. Ahmad et al. described a simple application by using a drop of Histoacryl^®^ glue followed by OmniGen^®^ to close a corneal perforation [211]. To be noted, these surgeries and methods above are usually applied for partial LSCD, but not for diffuse severe cases of LSCD [212]. 

### 5.3. Limbal Stem Cell Transplantation Techniques

Various techniques for limbal stem cell transplantation have been reported as a practical solution for ocular surface restoration in severe cases of LSCD [101]. According to the classification proposed by the International Corneal Society, the nomenclature for ocular surface stem cell transplantation was based on these parameters: anatomic source of tissue transplanted (conjunctival, mucosal, or keratolimbal); autologous or allogeneic (cadaveric or living-related), and cell culture techniques (cell culture of cadaveric limbal tissue) [213]. At the World Ophthalmology Congress Virtual 2020, Dua proposed that it is essential to establish whether the LSCD is active or not, limited or progressive, partial or total and unilateral or bilateral [214].

#### 5.3.1. Conjunctival-Limbal Autograft

The conjunctival-limbal autograft (CLAU) procedure is characterized by transplantation of autologous limbal tissue from the healthy contralateral eye, which is described as one of the successful strategies for restoring the corneal surface in unilateral cases of LSCD with no antigenic challenge [215]. In 1989, Kenyon and Tseng recommended limbal autograft transplantation to treat unilateral LSCD by presenting 26 consecutive acute and chronic LSCD cases [14]. The procedure of CLAU includes removal of the fibrovascular pannus from the diseased eye by peritomy and superficial keratectomy [14]. Two limbal grafts from the 6 and 12 o’clock positions of the healthy contralateral eye are harvested, transplanted and secured to the diseased eye [14]. The most common use of CLAU has been performed in surgery for pterygium or severe corneal chemical burn [216,217]. In recent years, several studies have reported the long-term ocular surface stability of CLAU in unilateral LSCD [218]. By reviewing patients with a minimum follow-up time of one year after CLAU, Eslani et al. showed that CLAU could successfully provide long-term ocular surface and vision stability in 77.8% of eyes in patients with unilateral LSCD [218].

However, one of the limitations of this procedure is that CLAU requires about one-third of the autologous limbal tissue from the healthy contralateral eye, which may cause potential risks of LSCD to the healthy eye [219,220,221]. Another limitation is that some questions remain as to the optimal size of the transplanted autologous limbal tissue for complete and stable visual outcomes [222,223,224]. Baradaran-Rafii et al. reported that one 60° block of CLAU as a graft is insufficient for stable epithelialization of the cornea resulting in progressive conjunctivalization [224]. Considering the limitations of the simple CLAU technique, CLAU combined with AMT effectively improves vision and maintains long-term ocular surface stability [225,226].

#### 5.3.2. Keratolimbal Allograft and Living-Related Conjunctival Limbal Allograft

CLAU is most successful in maintaining long-term ocular surface stability in unilateral LSCD. However, CLAU is not available in bilateral cases of LSCD. Thus, allograft transplantation techniques, such as keratolimbal allograft (KLAL) and living-related conjunctival limbal allograft (lr-CLAL) are the options to restore the ocular surface in bilateral LSCD [227]. In the KLAL procedure, limbal tissue attached to a corneoscleral carrier from a cadaveric donor is transplanted to the recipient eye, which provides a complete limbus for transplantation with a large load of LESCs [228,229]. KLAL is an ideal option for diseases, such as contact lens wear-related LSCD, iatrogenic LSCD and aniridia that minimally affect the conjunctiva [229]. The technique of KLAL is described in the protocol of the Minnesota Lions Eye Bank [230]. KLAL is often combined with other procedures, such as CLAU or lr-CLAL, to enhance success in ocular surface repair, while KLAL does not provide a healthy conjunctiva [231]. Biber et al. developed the Cincinnati procedure, a combined lr-CLAL and KLAL, to manage patients with unilateral severe ocular surface failure [231,232]. Chan et al. proposed the modified Cincinnati procedure (CLAU coupled with KLAL) in order to harvest a larger piece of donor tissue in patients with severe unilateral total ocular surface failure [232]. In 2017, Sepsakos et al. presented the first case of donor-derived transmission of melanoma that occurred after a KLAL in a 56-year-old woman with a history of LSCD [233]. After cessation of immunosuppression and removing the donor KLAL, the developed melanoma was wholly cured [233]. Concerning this case, the Eye Bank Association of America (EBAA) guidelines in 2016 were amended to include stricter vascularized ocular tissue transplantation parameters [233,234]. Based on Minimum Medical Standards, the European Eye Bank Association (EEBA) differentiates between vascular tissue donation and avascular tissue donation, and made restrictions for donors with a history of malignancy for vascularized tissue donations [233,235]. 

Nevertheless, KLAL may represent a challenge and bear an increased risk of failure in patients with severe corneal chemical injury, Stevens-Johnson syndrome, and mucous membrane pemphigoid [236,237,238]. Since allogenic grafts containing vascularized tissue are highly immunogenic, immunologic rejection is the most common postoperative complication after KLAL [239,240]. Hence, systemic immunosuppression is crucial to prevent immune rejection and chronic inflammation [241]. In 2003, Holland et al. reported that KLAL is effective in treating aniridic keratopathy, and the success rate was 90% in patients receiving systemic oral immunosuppressants (prednisone, cyclosporine A, and azathioprine) compared to 40% in patients treated only with topical immunosuppression (corticosteroid, cyclosporine A, and loteprednol etabonate 0.5%) [242]. Based on the knowledge obtained from solid organ transplantation, multidrug regimens are recommended with a higher level of immunosuppression and lower toxicity [241]. Krakauer et al. presented a series of patients with KLAL, who received systemic immunosuppression with prednisone, mycophenolate mofetil, and tacrolimus [241]. The authors concluded that systemic immunosuppression did not result in serious adverse effects and was relatively safe [241]. However, immunosuppression can cause persistent epithelial defects and increase the risk for infectious keratitis, especially fungal keratitis [243].

The lr-CLAL technique is usually applied to normal limbal tissue on a conjunctival carrier from a living relative, which is an alternative option in bilateral LSCD [244]. The surgical procedure of lr-CLAL is similar to CLAU [245]. Compared to KLAL, lr-CLAL provides a limited amount of tissue with fewer LESCs transplanted [245]. However, the advantage of lr-CLAL is to offer some degree of immune histocompatibility and to reduce the dosage of systemic immunosuppressives [245]. Human leukocyte antigen (HLA) and ABO typing are conducted preoperatively in the recipient and all consenting relatives, and the individual with the best match available is chosen as a donor [246]. In a retrospective, comparative, and interventional cohort study in 2020, the long-term outcomes of lr-CLAL and KLAL were compared in patients with LSCD [247]. The results showed that lr-CLAL presented lower rejection and higher graft survival rates compared with the KLAL procedure [247]. HLA-matched conjunctival limbal allograft and triple-agent systemic immunosuppression are effective in improving long-term survival [246,247]. Importantly, lr-CLAL bears the risk of immunologic rejection even in HLA-matched recipients, indicating that systemic immunosuppression may even span their survival time [248].

#### 5.3.3. Ex Vivo Cultivated Limbal Epithelial Transplantation

According to the source of the LESCs graft, ex vivo cultivated limbal epithelial transplantation (CLET) is divided into autologous CLET (auto-CLET) and allogeneic CLET (allo-CLET), which both have been used in clinical trials [249]. Compared to CLAU, KLAL, and lr-CLAL, the advantages of CLET are the requirement of a smaller amount of donor tissue, a shorter time for corneal epithelialization and lower risk of graft rejection [250,251]. In 1997, Pellegrini et al. cultivated limbal cells from a 1 mm^2^ biopsy sample from the limbus of the healthy eye to generate autologous corneal epithelial sheets and to restore the human corneal surface [252]. In the auto-CLET procedure of Pellegrini et al., 1–2 mm^2^ biopsy samples were harvested from the limbus from the patients’ healthy fellow eyes [252]. After biopsy, samples were cultured in vitro and grafts were mounted on a petrolatum gauze or a soft contact lens [252]. Cultured epithelial grafts were placed on the prepared eye and a soft therapeutic hydrophilic contact lens was then placed over the graft [252]. The study of Pellegrini et al. demonstrated that auto-CLET may offer a therapeutic option to patients with unilateral LSCD [252]. The presence of adequate numbers of ΔNp63α-positive stem cells in the graft has been demonstrated as an indicator for long-term clinical success [63]. Auto-CLET is considered an ideal surgical method used in unilateral LSCD to avoid the risk of immunological rejection [253]. In bilateral total LSCD cases, allo-CLET is used to be an effective measure that utilizes LESCs harvested from a cadaveric donor to reestablish corneal structure without the need of a biopsy [251]. A prospective and noncomparative case series showed that the clinical success rates were lower in the auto-CLET group (66.7%) than in the allo-CLET group (85.7%), since lid abnormalities were found in more eyes in the auto-CLET group (six of 12 eyes) than in the allo-CLET group (one of seven eyes) [251]. Shortt et al. applied the Clinical Outcome Assessment in Surgical Trials of Limbal stem cell deficiency (COASTL) tool to evaluate the 3-year outcomes for allo-CLET in patients with Stevens-Johnson syndrome or aniridia [254]. After systemic immunosuppression with oral cyclosporin or mycophenolate mofetil for 6 months, improvement in visual acuity was shown in 79% of eyes at 6 months, 71% at 12 months, 64% at 18 months, and 57% at both 24 and 36 months [254]. The prognosis of immune rejection after allo-CLET has been taken into consideration and investigated in patients [255]. Forty-two eyes of forty-one patients with total LSCD received allo-CLET, and all patients after surgery received systemic immunosuppressives with intravenous methylprednisolone (2 mg/kg) and oral prednisolone (1 mg/kg) [255]. The immune rejection rate at 6 months and during the follow-up of 17.8 ± 3.8 months after surgery was 4.76% (2/42 eyes) and 23.8% (10/42 eyes), respectively [255]. 

Currently, several techniques of CLET have been developed for the sake of minimizing the risk of depletion to the contralateral or donor limbus [256]. There are several types of scaffolds for cell expansion in vitro, such as human amniotic membranes, fibrin matrix, human anterior lens capsules, silk fibroin, and siloxane hydrogel contact lenses [256,257,258]. A bioengineered graft has been designed to seed cultured LESCs on a matrix derived from amniotic membrane [256]. However, since the supply of grafts (e.g. human amniotic membrane) is unreliable and requires expensive screening regimes, a tissue-engineered scaffold that can increase cultivation and transplantation efficacy of CLET will become a promising therapy direction to provide a safe platform for CLET [259]. Levis et al. described the use of plastic compressed collagen as a substrate for LESCs expansion and stratification into a corneal epithelial equivalent, which may provide a suitable alternative to amniotic membrane as a substrate for CLET [260]. In 2015, Holoclar® (Chiesi, Parma, Italy), the first commercial therapy based on LESCs expanded on fibrin scaffolds has been approved in Europe as the first commercially available stem cell therapy for unilateral LSCD [261]. In 2019, the recombinant human collagen type I (RHC I) and collagen-like peptide (CLP) hydrogels successfully supported the cultivation of LESCs by using a xeno-free cultivation protocol [262]. Real Architecture for 3D Tissue (RAFT), a tissue equivalent, is produced from biocompatible and low immunogenic type 1 collagen, capable of maintaining a barrier and protecting the underlying stroma [259]. Various tissue-engineered scaffolds for CLET have been studied with impressive success. Nevertheless, these tissue-engineered techniques require highly specialized cell expansion expertise, a sophisticated laboratory, and a significant investment of time and money [263].

#### 5.3.4. Simple Limbal Epithelial Transplantation

In 2012, a new transplantation method was reported by Sangwan et al. that required few donor tissue and no specialized equipment and infrastructure [264]. The novel surgical technique was termed simple limbal epithelial transplantation (SLET) and combines the benefits of CLAU and CLET [264]. In their first report, the authors performed SLET in six patients with unilateral LSCD [264]. In their surgical protocol, the authors took a 2 × 2 mm piece of donor limbal tissue from the healthy fellow eye and divided it into eight to ten small pieces [264]. These tiny limbal transplants were distributed and glued over an amniotic membrane placed on the cornea [264]. The outcomes of patients with autologous SLET (auto-SLET) for unilateral LSCD have been reported in 2016 across eight centers in three countries [265]. Of 68 eyes from 68 patients that received auto-SLET, 57 eyes (83.8%) achieved a completely epithelized and avascular corneal surface with a minimum of 6 months of follow-up [265]. In 2020, Shanbhag et al. compared clinical outcomes of CLAU, auto-CLET, and auto-SLET techniques in unilateral LSCD [266]. They found that the anatomical and functional success rates of auto-SLET (78%; 68.6%) and CLAU (81%; 74.4%) are better than those of CLET (61.4%; 53%) in 1023 eyes [266]. Since auto-CLET fails in around 20–30% of clinical cases, auto-SLET is indicated as an effective alternative method to CLET in eyes with recurrence of LSCD after CLET [267]. The results of auto-SLET combined with sequential penetrating keratoplasty in seven patients with unilateral LSCD showed visual success in four patients between 2012 and 2017 [268]. In 2021, eight eyes (8/10, 80%) which underwent auto-SLET kept a successfully regenerated stable corneal surface [269]. However, when severe bilateral LSCD occurs, auto-SLET is not accessible and available in patients. To achieve faster ocular restoration, allogeneic SLET (allo-SLET) has been applied in patients especially with severe bilateral LSCD, such as Stevens-Johnson syndrome and mucous membrane pemphigoid [270].

The difference between auto-SLET and allo-SLET is that in auto-SLET, grafts are taken from the contralateral eye of the patient, while in allo-SLET limbal grafts are obtained from deceased or living donors [271]. Like in other allogenic corneal transplantation techniques, patients who received allo-SLET need long-term systemic immunosuppression to improve graft survival [272]. Allo-SLET was reported in a 41-year-old woman after chemical injury [272]. Three months after surgery, allograft rejection was seen with peripheral corneal neovascularisation and acute pain [272]. After administration of pulse doses of intravenous methyl prednisolone with topical prednisolone acetate 1% eye drops, the symptoms resolved within one week [272]. Riedl et al. reported on clinical records of 14 patients treated with allo-SLET alone or combined with penetrating keratoplasty with limbal tissue from cultivated cadaveric donor eyes. The authors found that one year after transplantation, 71.4% of the eyes had a stable corneal epithelium suggesting that the technique is an alternative treatment procedure when autologous limbal tissue is not available [273]. Iyer et al. reported on records of 18 eyes of 17 patients, who underwent allo-SLET in the acute stage after chemical injury between 2013 and 2016 [274]. The authors showed that 17 of the 18 treated eyes (94.11%) achieved complete epithelialization in the immediate postoperative period [274]. However, over time seven eyes had a gradual failure of the allograft and 3 eyes (16.7%) had symblepharon formation involving one to two quadrants [274]. In 2021, the success rate of allo-SLET was 66.67% [269]. Jackson et al. reported that the success rate of allo-SLET is lower than that of auto-SLET, since immune rejection is unavoidable in allo-SLET [270].

The advantages of SLET compared to CLET are considered prominent, which allows the cell expansion to take place on the ocular surface rather than in a clinical-grade laboratory [271]. Moreover, SLET could have positive an influence on patients’ lives suffering from unilateral or bilateral chronic LSCD.

#### 5.3.5. Non-LESCs Transplantation

In the treatment of severe bilateral ocular surface disorders, e.g., Stevens-Johnson syndrome and ocular cicatricial pemphigoid, by allogenic limbal tissue transplantation techniques, allograft rejection, and side effects because of immunosuppressive treatment are the most severe clinical adverse events reducing the patients’ quality of life. Therefore, the concept and clinical application of an autologous mucosal epithelium of non-ocular origin as a substitute for the autologous epithelium of ocular origin has been considered as an alternative [275]. Cultivated oral mucosal epithelial transplantation (COMET) was developed to utilize the autologous epithelium of oral mucosa to restore a stable ocular surface [276,277]. Nakamura et al. showed by electron microscopic examination that an oral epithelial sheet cultivated on denuded amniotic membrane had junctional specializations (e.g., desmosomal, hemi-desmosomal, and tight junctions), which were similar to those of corneal epithelial cells in vivo [277]. According to this outcome, Nakamura et al. have concluded that oral epithelial cells are suitable for ocular surface reconstruction [277]. In 2004, Nakamura et al. first performed the transplantation of cultivated autologous oral epithelial cells in patients with bilateral LSCD [276]. Autologous oral epithelial cells were cultured ex vivo for 2–3 weeks on human amniotic membrane with a 3T3 fibroblast co-culture and an air-lifting method [276]. The cultivated oral epithelial sheet was transplanted onto the damaged ocular surface, and the entire corneal surface was free of epithelial defects 48 h after transplantation [276]. In a long-term follow-up of COMET by Prabhasawat et al., in vivo confocal microscopy revealed a cornea-like phenotype, and impression cytology with immunofluorescence staining showed positivity for CK3 and CK12 in six of 13 eyes [278]. In contrast, seven eyes showed mostly a conjunctival phenotype [278]. MUC5AC as a conjunctival goblet cell marker was found only in failed cases [279,280]. In a retrospective cohort study from 2002 to 2008, COMET had a success probability of 79.6% for overall fornix-reconstruction, of 100% for thermal/chemical injury and of 53.3% for ocular cicatricial pemphigoid, respectively, at five years postoperatively [281]. In a prospective interventional case series from 2013 to 2017, which included eyes that received COMET because of chronic Stevens-Johnson syndrome sequelae, 82.2% (37/45) of eyes had an improvement in visual acuity, 13.3% (6/45) presented with no change, whereas 4.4% (2/45) of eyes had worsening of visual acuity [282]. Cabral et al. summarized 14 studies on COMET, which obtained tissue samples from the buccal mucosa and two studies that obtained tissue from the lip from 2004 to 2019 [283]. Based on these studies, 70.8% (172/243) of eyes with LSCD achieved a stable epithelium, and 68.2% (225.6/331) of eyes had some visual improvement based on publications from 2004 to 2019 [283]. Taken together, COMET is considered a promising therapy for bilateral LSCD [283].

In addition, multi-potential mesenchymal stem cells (MSCs) from adult bone marrow have the multilineage potential to differentiate to various types of cells under different in vitro conditions, including cardiac cells, epithelial cells, endothelial cells, fat cells, nerve cells, and bone marrow stromal cells [284,285,286]. Jiang et al. isolated and purified rat MSCs by using a gradient isolation procedure and used rat corneal stromal cells in a transwell co-culture system to induce these cells [285]. The results showed that MSCs induced by corneal stromal cells could transdifferentiate into corneal epithelial cells in vitro, which had remarkable effects on the reconstruction of the corneal surface of rats [285]. Even though MSCs can acquire specific characteristics of corneal epithelial cells, subcutaneous adipose tissue (AT) is more easily obtained from liposuction aspirates than MSCs [287]. Extraocular human AT-derived MSCs (AT-MSCs) have been shown to acquire some features of corneal epithelial-like cells in vitro, which could provide a novel alternative autologous cell source for patients with bilateral LSCD [287]. AT-MSCs present a paracrine action via suppressing secretion of trophic factors and modulating the inflammation and immune reaction to benefit the regenerative processes [286,288]. Priming human AT-MSCs with limbal stem cell specific medium may potentiate their ability to decrease neovascularization and inflammation in LSCD [288]. AT-MSC sheets with positive expression of corneal epithelial markers K3 and K12 were developed to facilitate effective delivery of these cells to the damaged site, suggesting that AT-MSCs combined with cell sheet technology may become a novel therapy in treating LSCD [289]. It has been shown that MSCs play a protective role in corneal repair by secreting extracellular nano-sized vesicles mainly composed of ectosomes and exosomes, which enable the transfer of microRNA to target cells. [290]. Another study reported that thrombospondin-1 ameliorated hypoxia-induced paraptosis and promoted wound healing and remodeling by regulating exosomal protein expression in human corneal epithelial cells [291]. However, exosomes derived from antigen presenting cells, such as dendritic cells and B lymphocytes, can carry major histocompatibility complex class I and II molecules on the surface, which can induce stronger antigen-specific immune responses [292]. Moreover, it is still a challenge to isolate and purify exosomes in vitro [293].

Furthermore, based on various other cell-based therapies of LSCD, murine vibrissae hair follicle bulge-derived stem cells (HFSCs) have been reported to have therapeutic potential for reconstructing the ocular surface in 80% of the transplanted animals [294]. However, the number of published studies with HFSCs are limited. Human immature dental pulp stem cells (hIDPSCs) express similar key characteristics as LESCs, such as ABCG2, integrin β1, p63, Cx43, and K3/12, indicating a potential alternative cell source for corneal reconstruction [295]. Moreover, inorganic polyphosphate (polyP) was demonstrated to increase cell viability/growth and migration of corneal epithelial cells, providing a potential biomaterial to treat LSCD [296]. Wang et al. suggested that the water-soluble Na-polyP can be used as a biomimetic tear fluid to restore the corneal surface [296]. Except from polyP, many other substances are reported to have potential functions in improving corneal regeneration, such as trehalose, polymer 2-methacryloyloxyethyl phosphorylcholine (poly-MPC), glycoprotein 340, ferrostatin-1, agrin, fiber-reinforced gelatin methacrylate hydrogel, and keratan sulfate [297,298,299,300,301,302]. These potential substances may exert beneficial effects in clinical practice for patients with LSCD. 

## 6. Conclusions

In this review, we provided an overview on the location and markers of LESCs as well as on and the pathophysiology and therapy of LSCD. LESCs and the limbus’s microenvironment are essential to maintain the corneal immune privilege [159]. It is challenging to choose therapeutic options for patients with LSCD, which depends on the etiology, the degree of severity, and the laterality of LSCD. Therefore, we reviewed the advantages and limits of the clinically established conservative measures, such as autologous serum, anti-inflammatory drugs, and polyphosphate as well as surgical techniques (CLAU, KLAL, lr-CLAL, CLET, and SLET). In cases, in which autologous limbal tissue is not available for transplantation, such as cases with severe bilateral LSCD, allogenic tissue needs to be utilized. In such cases, allograft rejection may be one of the most severe side-effects, thus, requiring long-term immunosuppressive treatment. New promising materials and methods, which are currently in the experimental stage, may be helpful in improving corneal epithelial regeneration. 

## Figures and Tables

**Table 1:** Corneal keratins in the development and wound healing of corneal and limbal epithelia.

Keratins	Species	Stage	Location	References
K3	Rabbit	21-day embryos	The peridermal layer of the cornea	[98]
		23-day embryos	The suprabasal layer of the cornea	
		7 to 12 days after birth	The basal layer of the cornea	
		Adult	The basal layer of the corneal epithelium and the suprabasal layer of the limbal epithelium	
K4	Mouse	16-, 18-, and 20-day embryos 2 and 4 days after birth	The superficial layer of the cornea	[94]
		Adult	Not observed	
K5/K12	Mouse	7 days after birth	The basal and apical cells of the central and peripheral cornea and limbus	[95]
K12	Rabbit	17-day embryos	The peridermal layer of the cornea	[98]
		23-day embryos	The basal layer of the cornea	
		Adult	The basal layer of the corneal epithelium and the suprabasal layer of the limbal epithelium	
K12	Chick	12-day embryos	Both peridermal and basal ectodermal layers of the cornea	[98]
		14- to 21-day embryos	All the epithelial strata of the central cornea	
		21-day embryos	The suprabasal layer of the limbal epithelium	
K12	Mouse	15-day embryos	The superficial layer of the corneal epithelium	[94]
		18-day embryos	The suprabasal layer of the cornea	
		0 h after wounding	The peripheral corneal epithelium	[97]
		24 h after wounding	Occasionally in superficial cells of the central and peripheral cornea	
K14	Mouse	15-day embryos	Corneal epithelial cells	[94]
		7 days after birth	The basal layers over the entire mouse ocular surface with higher expression at the limbal region compared with the central cornea	[95]
		49 days after birth	The localization decreases in the central corneal epithelium but remains strong at the limbus.	
		0 h after wounding	Restricted to the limbus	[97]
		24 h after wounding	The layer of epithelial cells that covered the defect	
		1 day after wounding	Corneal epithelial cells at and behind the leading edge	[95]
		7 days after wounding	The corneal center	
		28 days after wounding	Cells adjacent to an erosion at the corneal center and around goblet cell clusters at the limbus	
K15	Mouse	7 days after birth	Limbal cells and well spread apical cells of the corneal periphery and center	[95]
		49 days after birth	The localization decreases in the central corneal epithelium but remains strong at the limbus.	
		1 day after wounding	Extending toward the leading edge	
		7 days after wounding	The corneal center	
		28 days after wounding	Cells adjacent to an erosion at the corneal center and around goblet cell clusters at the limbus	
K17	Human	Within 24 h (for ex vivo) to 48 h after death	Clusters of limbal basal cells in normal corneas and significantly decreased in diabetic limbal basal epithelium	[99]
K18	Mouse	14 days after birth	The limbus	[95]
		7 days after wounding,	The center of the clusters	
		28 days after wounding	Compound niches	
K8/K19	Mouse	7 days after birth	Basal and suprabasal cells throughout the corneal epithelium and limbus	[95]
		14 days after birth	Fewer cells on the central cornea and more restricted toward the peripheral and limbal region	
		1 day after wounding	K19^ +^ clusters are migrating away from the limbus	
		7 days after wounding	K19 is localized to cells at the edges of large clusters	
		28 days after wounding	Near the limbal region and on the peripheral cornea	[95,96]

**Table 2:** Etiologies of LSCD.

Congenital	Acquired
Congenital aniridia [112,113]	Stevens-Johnson syndrome [114]
Peter’s anomaly [115]	Toxic epidermal necrolysis [116,117]
Ectrodactyly-ectodermal-dysplasia-clefting syndrome [118]	Graft-versus-host disease [119]
Keratitis-ichthyosis-deafness syndrome [120]	Ocular cicatricial pemphigoid [121,122]
Dyskeratosis congenita [123,124]	Conjunctival intraepithelial neoplasia [125,126]
Multiple endocrine deficiency [127,128]	Corneal intraepithelial neoplasia [129]
Congenital erythrokeratodermia [130,131]	Pterygium [132]
Xeroderma pigmentosum [133,134]	Chemical burn [135]
Turner syndrome [136]	Thermal injury [137]
	Mustard gas [138,139]
	Trachoma [140]
	Herpetic Keratitis [141]
	Ocular surgeries [142,143]
	Contact lens wear [107,144,145]
	Cryotherapy [146,147]
	Medication toxicity (mitomycin C, 5-fluorouracil, and preservative) [148,149,150,151,152]
	Radiation therapy [153]
	Phototherapeutic keratectomy [154]
	Intravitreal injections [155]
	Neurotrophic keratitis [156,157]
	Bullous keratopathy [158]

Abbreviations: LSCD: limbal stem cell deficiency.

## Data Availability

Not applicable.

## Abbreviations:

List of abbreviations

**Abbreviation**	**Definition**
AT	adipose tissue
allo-CLET	allogeneic CLET
allo-SLET	allogeneic SLET
ACER	amnion-assisted conjunctival epithelial redirection
AMT	amniotic membrane transplantation
AT-MSCs	AT-derived MSCs
ABC	ATP binding cassette
auto-CLET	autologous CLET
auto-SLET	autologous SLET
BCRP1	breast cancer resistance protein 1
BrdU	bromodeoxyuridine
CLAU	conjunctival-limbal autograft
Cx43	connexin 43
COMET	cultivated oral mucosal epithelial transplantation
EGFR	epidermal growth factor receptor
CLET	ex vivo cultivated limbal epithelial transplantation
HFSCs	hair follicle bulge-derived stem cells
hIDPSCs	human immature dentalpulp stem cells
IL	interleukin
KGF	Keratinocyte growth factor
KGFR	keratinocyte growth factor receptor
KLAL	keratolimbal allograft
LESCs	limbal epithelial stem cells
LSCD	limbal stem cell deficiency
lr-CLAL	living-related conjunctival limbal allograft
MSCs	mesenchymal stem cells
ΔNp63α	nuclear p63 transcription factor
PlGF	placenta growth factor
polyP	polyphosphate
poly-MPC	polymer 2-methacryloyloxyethyl phosphorylcholine
PDL	pre-Descemet’s layer
RHCIII	recombinant human collagen type III
SSCE	sequential sector conjunctival epitheliectomy
SP	side population
SLET	simple limbal epithelial transplantation
SAoO	Swiss Academy of Ophthalmology
TAC	transit amplifying cell
VEGF	vascular endothelial growth factor
VEGFR1	VEGF receptor 1
